# Disrupted functional connectivity between sub-regions in the sensorimotor areas and cortex in migraine without aura

**DOI:** 10.1186/s10194-020-01118-1

**Published:** 2020-05-06

**Authors:** Zhaoxia Qin, Jingjing Su, Xin-Wei He, Shiyu Ban, Qian Zhu, Yangyang Cui, Jilei Zhang, Yue Hu, Yi-Sheng Liu, Rong Zhao, Yuan Qiao, Jianqi Li, Jian-Ren Liu, Xiaoxia Du

**Affiliations:** 1 Shanghai Key Laboratory of Magnetic Resonance and Department of Physics, School of Physics and Electronic Science, East China Normal University, 3663 North Zhong-Shan Road, 200062 Shanghai, People’s Republic of China; 2 Department of Neurology and Jiuyuan Municipal Stroke Center, Shanghai Ninth People’s Hospital, Shanghai Jiao Tong University School of Medicine, 639 Zhizaoju Road, 200011 Shanghai, People’s Republic of China; 3 Clinical Research Center, Shanghai Jiao Tong University School of Medicine, Shanghai, 200011 China; 4 Clinical Science, Philips Healthcare, Shanghai, 200040 P. R. China

**Keywords:** Migraine, Sensorimotor, Sub-region, Resting state, Functional MRI, Functional connectivity

## Abstract

**Background:**

Migraine is a severe and disabling brain disorder, and the exact neurological mechanisms remain unclear. Migraineurs have altered pain perception, and headache attacks disrupt their sensory information processing and sensorimotor integration. The altered functional connectivity of sub-regions of sensorimotor brain areas with other brain cortex associated with migraine needs further investigation.

**Methods:**

Forty-eight migraineurs without aura during the interictal phase and 48 age- and sex-matched healthy controls underwent resting-state functional magnetic resonance imaging scans. We utilized seed-based functional connectivity analysis to investigate whether patients exhibited abnormal functional connectivity between sub-regions of sensorimotor brain areas and cortex regions.

**Results:**

We found that patients with migraineurs without aura exhibited disrupted functional connectivities between the sensorimotor areas and the visual cortex, temporal cortex, posterior parietal lobule, prefrontal areas, precuneus, cingulate gyrus, sensorimotor areas proper and cerebellum areas compared with healthy controls. In addition, the clinical data of the patients, such as disease duration, pain intensity and HIT-6 score, were negatively correlated with these impaired functional connectivities.

**Conclusion:**

In patients with migraineurs without aura, the functional connectivities between the sensorimotor brain areas and other brain regions was reduced. These disrupted functional connectivities might contribute to abnormalities in visual processing, multisensory integration, nociception processing, spatial attention and intention and dysfunction in cognitive evaluation and modulation of pain. Recurrent headache attacks might lead to the disrupted network between primary motor cortex and temporal regions and between primary somatosensory cortex and temporal regions. Pain sensitivity and patient quality of life are closely tied to the abnormal functional connectivity between sensorimotor regions and other brain areas.

## Introduction

Migraine is a severe and disabling brain disorder that is characterized by recurrent attacks of headache and multiple sensory symptoms [1, 2]. Multisensory integration of somatosensory, visual, auditory and olfactory stimuli by the migraine brain may be important for understanding migraine [3]. Migraineurs have altered pain perception during attacks [1], and headache attacks might affect their sensory information processing and sensorimotor integration [4].

The motor and premotor cortex (PMC) and S1 are supposed as constituting parts of the spatial discrimination and pain intensity pathways [5]. Moreover, the motor areas, the PMC, the supplementary motor area (SMA) and the anterior cingulate cortex (ACC) constitute a module that contributes to response selection and the generation of sensory information [6]. Anatomically, first-order thalamus neurons located in the ventral posteromedial nucleus project mainly to the trigeminal areas of S1 as well as of the second somatosensory cortex (S2) and the insular cortices [7]. In addition, the S1, primary motor cortex (M1), and insular cortex have been implicated in the ascending trigemino-thalamo-cortical nociceptive pathway [8].

A previous study found functional abnormalities in the S1 and right PMC in patients with MWoA [4]. Episodic migraineurs showed significant hypometabolism in the PMC and S1 relative to controls [9]. Connectivity between the left ventral striatum and ipsilateral preSMA was decreased in chronic migraine with medication-overuse headaches compared with chronic migraine alone [10]. A meta-analysis of excitatory primary motor cortex (M1) stimulation showed significant effects on reducing headache intensity and frequency of headache attacks in patients with migraine with a large effect size [11]. Migraine without aura showed decreased functional connectivity between the left hippocampus and contralateral SMA and bilateral inferior parietal gyri (IPG) [12]. Patients with a higher frequency of migraine attacks showed increased periaqueductal gray matter (PAG) connectivity with the S1 face representation area and the SMA, an area involved in pain expectancy [13].

The altered functional connectivity of sensorimotor regions with other brain areas would affect the multisensory integration and perception and processing of pain, and the intrinsic FC between sub-regions of sensorimotor areas and the cortex associated with migraine without aura needs further investigation. We speculate that the interactions between sensorimotor regions and multiple cortical areas are necessary for the integration of information within and across the sensory modalities and, thus, could play an important role in the initiation of migraine attack and/or the development of its associated symptoms. Mayka et al. developed the Human Motor Area Template (HMAT), which used outcomes from ALE analysis in combination with previously suggested anatomical guidelines and can be used for seed-based functional connectivity analysis [14]. In this study, we tested the following hypotheses. 1) The intrinsic FCs of the sub-regions of sensorimotor areas to many important brain regions in patients with migraine without aura (MWoA) may be altered. 2) These altered sensorimotor region connectivity patterns may be associated with features of disease severity, such as disease duration, pain intensity, attack frequency, and the Migraine Disability Assessment Scale (MIDAS) and Headache Impact Test (HIT-6) scores.

## Methods

### Subjects

Forty-eight patients with MWoA (mean ± SD age = 38.1 ± 10.4 years) and 48 healthy controls (HCs) (mean ± SD age = 39.0 ± 11.0 years) were enrolled from the outpatient clinic of the Department of Neurology at Shanghai Ninth People’s Hospital. Patients were diagnosed with MWoA by a neurologist based on the International Classification of Headache Disorders 3rd edition criteria [2]. Demographic and clinical data were registered and evaluated in our headache database, including age, sex, disease duration, attack frequency (times/month), attack duration (hours) and pain intensity of migraine attacks assessed by a visual analogue scale (VAS) [15]. Patients also completed the MIDAS [16] and HIT-6 [17] regarding the accurate assessment of their headache-related disability. All patients with MWoA were scanned during an interictal period, with no headache 48 h before or 24 h after MRI scans, and they did not report a migraine attack or discomfort during the MRI scans. Migraineurs reported that they did not take preventive medication and did not suffer chronic migraine. The HCs had no headaches or chronic pain disorders in the past year. Moreover, the immediate family members of the HCs did not suffer from migraine or other headaches. Other exclusion criteria were the following: left-handedness, drug abuse, any neurological or psychiatric diseases, metabolic diseases (e.g., diabetes mellitus) or cardiovascular diseases based on clinical examination and structured interviews. The demographic and clinical data are provided in Table 1.

**Table 1:** Demographic data and clinical scores of the MWoA group and control group

	Migraine group	Control group	
	(Mean ± SD)	(Mean ± SD)	*P* value
N	48	48	1
Sex (male)	29.2%	29.2%	1
Age (years)	38.1 ± 10.4	39.0 ± 11.0	0.68
Disease duration (years)	8.5 ± 6.0	–	–
Attack duration (hours)	15.3 ± 18.4	–	–
Attack frequency (times/months)	3.8 ± 3.3	–	–
Pain intensity VAS score	7.2 ± 1.8	–	–
MIDAS score	23.1 ± 28.6	–	–
HIT-6 score	60.4 ± 12.0	–	–

*VAS* visual analogue scale, *MIDAS* migraine disability assessment scale, *HIT-6* headache impact test, - No data

### MRI acquisition

MRI was carried out in a 3.0 Tesla Siemens Trio Tim system with a 12-channel head coil at the Shanghai Key Laboratory of Magnetic Resonance (East China Normal University, Shanghai, China). All subjects’ head movements were minimized with a Siemens dedicated filler. T1-weighted anatomical images were collected using a 3-dimensional magnetization-prepared rapid-acquisition gradient-echo pulse sequence (repetition time = 2530 ms, echo time = 2.34 ms, inversion time = 1100 ms, flip angle = 7°, number of slices = 192, sagittal orientation, field of view = 256 × 256 mm^2^, matrix size = 256 × 256, slice thickness = 1 mm, 50% gap). Following this, subjects were asked to remain motionless, keep their eyes closed, and stay awake and relaxed. Resting-state images consisted of 210 volumes of a T2*-weighted gradient-echo echo planar imaging pulse sequence (33 slices of 3.5 mm thickness, repetition time = 2000 ms, echo time = 30 ms, flip angle = 90°, transverse orientation, field of view = 220× 220 mm^2^, matrix size = 64 × 64, 25% distance factor).

### Data preprocessing

Functional data were preprocessed using the Data Processing Assistant for Resting-State fMRI (DPARSF, http://rfmri.org/DPARSF) and analyzed with Statistical Parametric Mapping (SPM12, http://www.fil.ion.ucl.ac.uk/spm). To avoid scanner instability, the first 10 volumes were discarded. The images for each subject were motion-corrected, and in no subjects was there significant movement (> 2 mm in any direction). The data from all the subjects were used for the subsequent analysis. We utilized the Friston [18] 24-parameter model to regress out the head motion effects from realignment. Functional volumes were slice-timing corrected and coregistered to the individual structural images without resampling. The transformed structural images were segmented [19] and normalized to the Montreal Neurological Institute (MNI) space. Based on the same deformation field, functional images were then normalized to the MNI space (3 × 3 × 3 mm^3^) using the Diffeomorphic Anatomical Registration Through Exponentiated Lie Algebra (DARTEL) tool [20]. Spatial smoothing was performed on the functional images by a 6 mm FWHM Gaussian filter. We performed temporal bandpass filtering (0.01 < f < 0.1 Hz), and the mean signals from the white matter, cerebrospinal fluid, and linear trends were also included as covariates to reduce the influence of low-frequency drift and high-frequency respiratory and cardiac effects.

### Seed-based FC

The mesial premotor cortex including pre-supplementary motor area (preSMA) and supplementary motor area proper (SMA), lateral premotor cortex including dorsal premotor cortex (PMd) and ventral premotor cortex (PMv), and primary sensorimotor cortex including primary motor cortex (M1) and primary somatosensory cortex (S1) have been identified as key cortical areas for sensorimotor function [14].The HMAT ROIs included M1, S1, the SMA, the preSMA, the PMd, and the PMv. We calculated Pearson’s correlation coefficients of the mean time courses extracted from the HMAT ROIs with the signals of whole-brain voxels. To improve the normality of the data distribution, we converted FC maps to FC Z-value maps by Fisher’s Z-transformation. Then, the voxelwise two-sample t-test with sex and age as covariates was conducted within a brain mask. The two-tailed (voxel-level) threshold was *p* < 0.001 in combination with familywise error (FWE) corrected to *p* < 0.05 at cluster level. Surviving clusters were deemed significant. We performed a normal distribution test and found that the clinical data did not conform to the normal distribution, so we used Spearman correlation test. Then individual mean z-values for the surviving clusters were extracted to determine Spearman’s correlation correlations with clinical data features, including disease duration, attack frequency, and attack duration, as well as VAS, MIDAS and HIT-6 scores. Thresholds were *p* < 0.05, one-tailed, and FDR corrected for multiple comparisons.

## Results

### Demographic and clinical characteristics

The demographic and clinical data of the migraine and control groups are presented in Table 1. The ages and gender proportions did not differ between the two groups.

### Seed-based FC

**Fig. 1:**
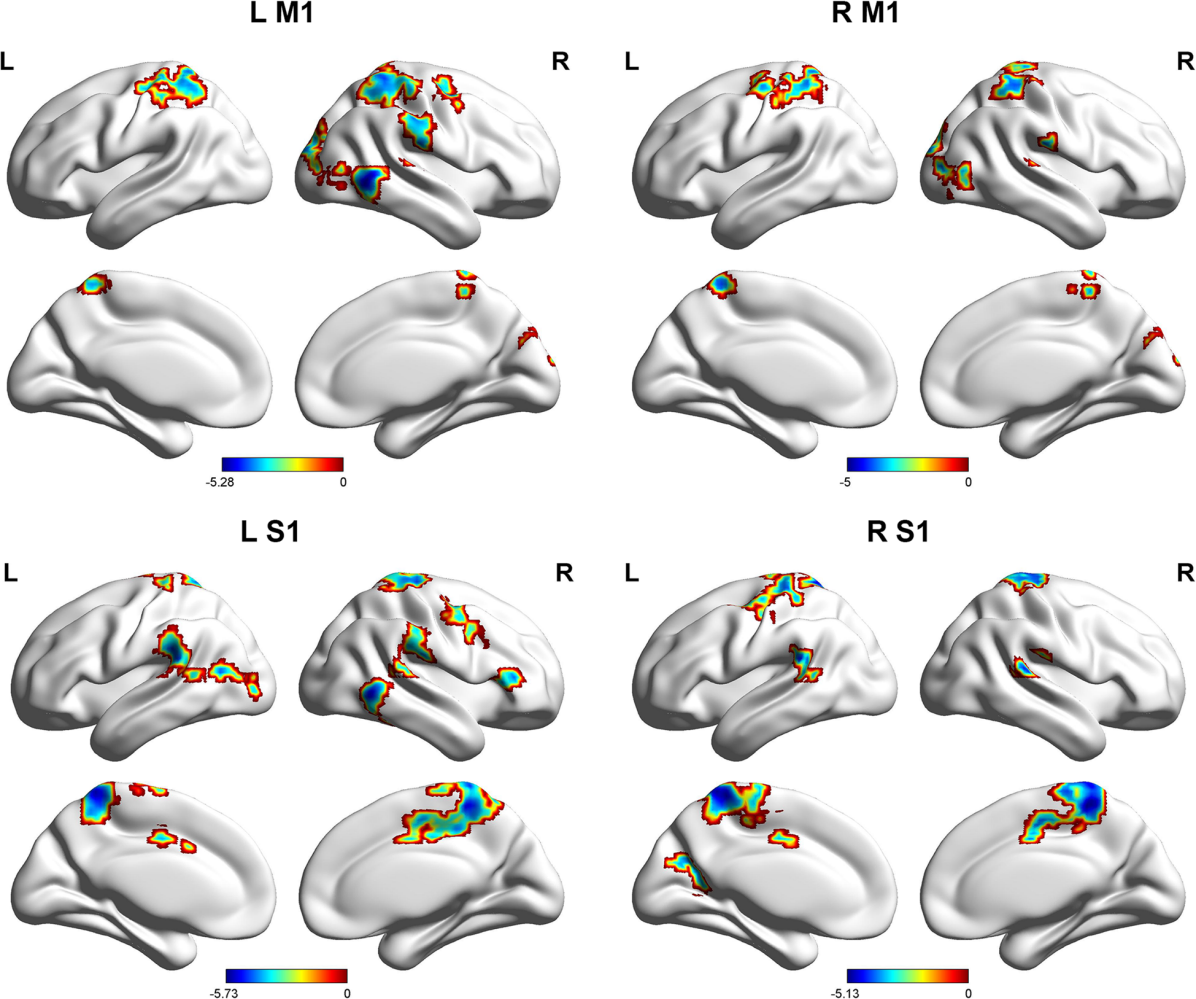
Sagittal views of the MNI brain areas showing reduced intrinsic functional connectivity (FC) with the M1 and S1 in MWoAs compared to healthy controls. The surviving clusters were assigned thresholds at *p* < 0.001 (voxel level) and were FWE corrected to *p* < 0.05 at the cluster level

The patients with MWoAs showed weaker FCs between L M1 and the right middle occipital gyrus, right middle temporal gyrus, right superior temporal gyrus, left superior parietal gyrus, right postcentral gyrus, and right middle frontal gyrus (Table 2, Fig. 1). We also found decreased FCs between R M1 and the right middle occipital gyrus, right superior temporal gyrus, left superior parietal gyrus, right postcentral gyrus, and right cuneus in patients with MWoAs relative to HC (Table 2, Fig. 1).

**Table 2:** Brain regions with decreased functional connectivity (FC) in migraine without aura (MWoA) compared to healthy controls (HC)

Predominant regions in the cluster	Cluster size	Peak T	MNI coordinates			*p*_*FWE* − *corr*_
	(number of voxels)		x	y	z	
Seed in L M1						
Right middle occipital gyrus	199	−5.28	36	−69	3	0.000
Right middle temporal gyrus	151	−5.19	57	−57	0	0.000
Right superior temporal gyrus	177	−4.85	63	−27	15	0.000
Parietal_Sup_L (aal)	303	−4.73	−30	− 48	57	0.000
Right postcentral gyrus	342	−4.51	30	−45	66	0.000
Right middle frontal gyrus	135	−4.44	36	−3	48	0.001
Seed in R M1						
Right middle occipital gyrus	122	−5.00	36	−69	6	0.002
Right superior temporal gyrus	60	−4.59	63	−27	15	0.046
Parietal_Sup_L (aal)	307	−4.57	− 30	− 45	57	0.000
Postcentral_R (aal)	164	−4.24	33	−42	57	0.000
Right cuneus	64	−3.97	21	−93	18	0.036
Seed in L S1						
Precuneus_L (aal) extending to	176 (971)	−5.73	−9	−48	69	0.000
Bilateral postcentral gyrus	229 (971)					
Bilateral Paracentral lobule	210 (971)					
Precuneus_R (aal)	112 (971)					
Right middle temporal gyrus	144	−5.25	57	−54	0	0.001
Right superior temporal gyrus	195	−4.96	63	−30	15	0.000
Left inferior parietal lobule	157	−4.84	−57	−39	21	0.000
Left middle temporal gyrus	108	−4.58	−45	−66	12	0.004
Right inferior frontal gyrus	61	−4.54	48	33	9	0.047
Postcentral_L (aal)	76	−4.22	−21	−30	66	0.020
Right middle frontal gyrus	112	−4.22	45	0	45	0.003
Seed in R S1						
Precuneus_R (aal) extending to	166 (1219)	−5.13	12	−42	63	0.000
Precuneus_L (aal)	98 (1219)					
Right cingulate gyrus	67 (1219)					
Bilateral postcentral gyrus	320 (1219)					
Bilateral paracentral lobule	252(1219)					
Bilateral precentral gyrus	246(1219)					
Right superior temporal gyrus	86	−4.78	63	−30	15	0.011
Left inferior parietal lobe	61	−4.59	− 60	− 42	21	0.046
Occipital_Sup_L (aal)	87	−4.13	−21	−60	18	0.010
Seed in L SMA						
Precentral_L (aal) extending to	95 (665)	−5.62	−51	0	42	0.000
Left precuneus	112 (665)					
Parietal_Inf_L (aal)	210 (665)					
Postcentral_L (aal)	198 (665)					
Right middle frontal gyrus extending to	131 (852)	−5.55	36	−6	51	0.000
Right inferior parietal lobule	145 (852)					
Postcentral_R (aal)	274 (852)					
Precentral_R (aal)	177 (852)					
Right cuneus	217 (605)	−5.22	15	−78	30	0.000
Left middle temporal gyrus	77 (605)					
Right superior temporal gyrus	76	−4.95	63	−27	15	0.020
Right middle temporal gyrus	267	−4.72	60	−54	6	0.000
Seed in R SMA						
Right middle frontal gyrus extending to	104 (1942)	−5.57	39	−6	51	0.000
Bilateral postcentral gyrus	373 (1942)					
Bilateral precentral Gyrus	330 (1942)					
Bilateral inferior parietal lobule	243 (1942)					
Bilateral cuneus	232 (1942)					
Bilateral precuneus	221 (1942)					
Right middle temporal gyrus	91	−4.28	57	−54	−6	0.009
Seed in R preSMA						
Left precuneus	110	4.05	−18	−72	39	0.004
Seed in R PMd						
Left precuneus	306	−4.88	−18	−57	54	0.000
Left middle occipital gyrus	200	−4.75	−36	−78	9	0.000
Cerebellum_8_R	83	−4.49	18	−60	−57	0.013
Right cuneus	144	−4.47	21	−93	18	0.001
Right middle occipital gyrus	103	−4.11	36	−81	0	0.005
Right postcentral gyrus	101	−4.11	9	−45	66	0.005
Left cuneus	68	−3.84	− 18	− 87	18	0.031
Seed in L PMv						
Left precentral gyrus	107	−4.50	−27	−30	57	0.004
Right cingulate gyrus	111	−3.92	−9	−9	39	0.003
Right postcentral gyrus	75	−3.77	12	−45	69	0.022
Seed in R PMv						
Bilateral precuneus extending to	139 (469)	−4.75	12	−45	63	0.000
Bilateral postcentral gyrus	173 (469)					
Supp_Motor_Area_L (aal)	109	−4.32	0	−18	51	0.004

The results were assigned thresholds at *p* < 0.001 (voxel level) and FWE corrected to *p* < 0.05 at the cluster level

The patients with MWoAs showed weaker FCs between L S1 and the bilateral precuneus, bilateral postcentral gyrus, bilateral paracentral lobule, bilateral middle temporal gyrus, right superior temporal gyrus, left inferior parietal lobule and right inferior frontal gyrus and right middle frontal gyrus (Table 2, Fig. 1). We also found decreased FCs between R S1 and the bilateral precuneus, right cingulate gyrus, bilateral postcentral gyrus, bilateral paracentral lobule, bilateral precentral gyrus, right superior temporal gyrus, left inferior parietal lobe, and left occipital superior gyrus (Table 2, Fig. 1).

**Fig. 2:**
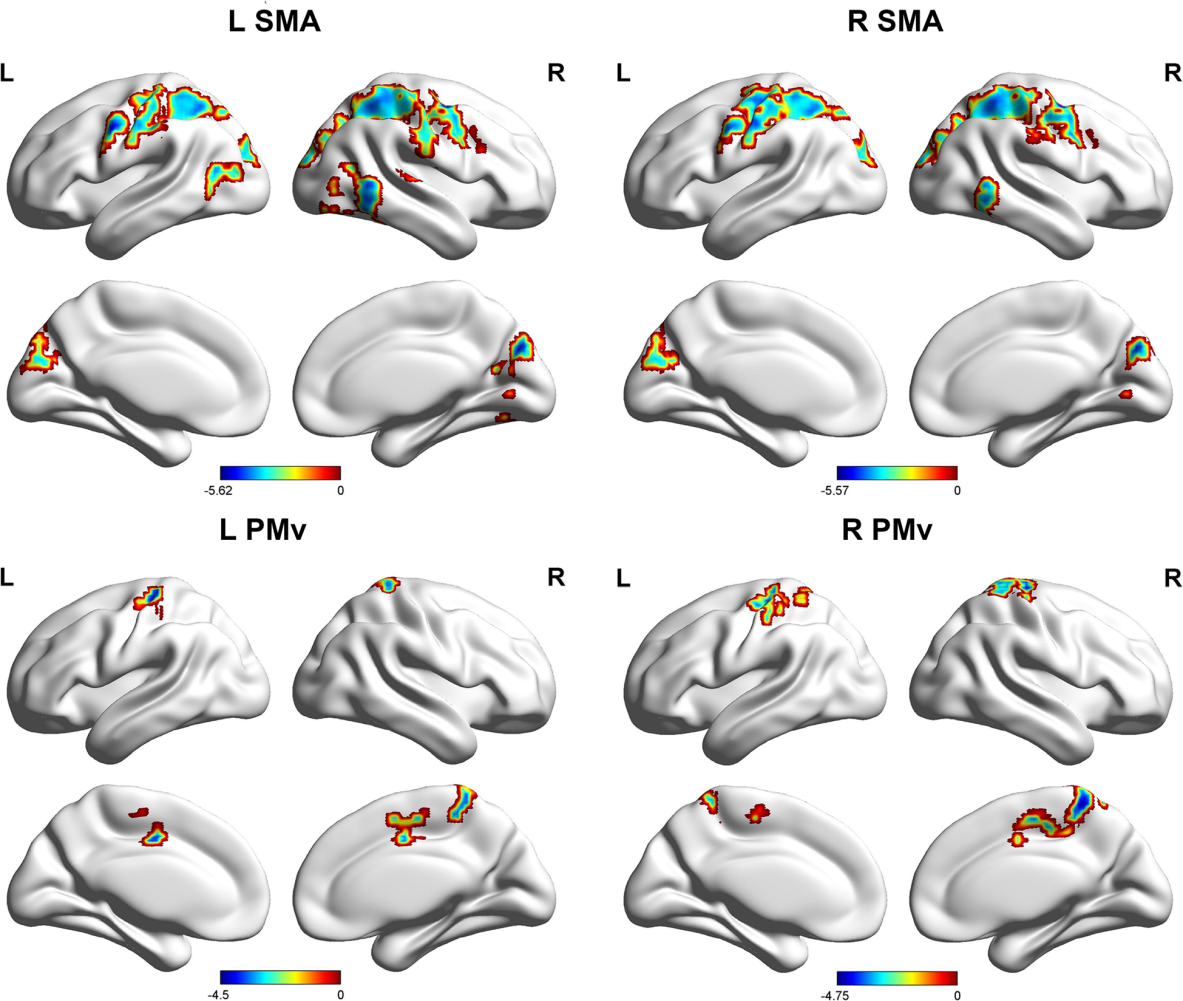
Sagittal views of the MNI brain areas showing reduced intrinsic functional connectivity (FC) with the SMA and PMv in MWoAs compared to healthy controls. The surviving clusters were assigned thresholds at *p* < 0.001 (voxel level) and were FWE corrected to *p* < 0.05 at the cluster level

The patients with MWoAs showed weaker FCs between the L SMA and bilateral precentral gyrus, bilateral postcentral gyrus, left precuneus and bilateral inferior parietal lobule and right middle frontal gyrus, right cuneus, bilateral middle temporal gyrus, and right superior temporal gyrus (Table 2, Fig. 2). The patients with MWoAs showed weaker FCs between the R SMA and the right middle frontal gyrus, bilateral postcentral gyrus, bilateral inferior parietal lobule, bilateral cuneus, bilateral precuneus, and right middle temporal gyrus (Table 2, Fig. 2).

The patients with MWoAs showed weaker FCs between the L PMv and the left precentral gyrus, right cingulate gyrus, and right postcentral gyrus (Table 2, Fig. 2). The patients with MWoAs showed weaker FCs between the R PMv and the bilateral precuneus, bilateral postcentral gyrus and L SMA (Table 2, Fig. 2).

**Fig. 3:**
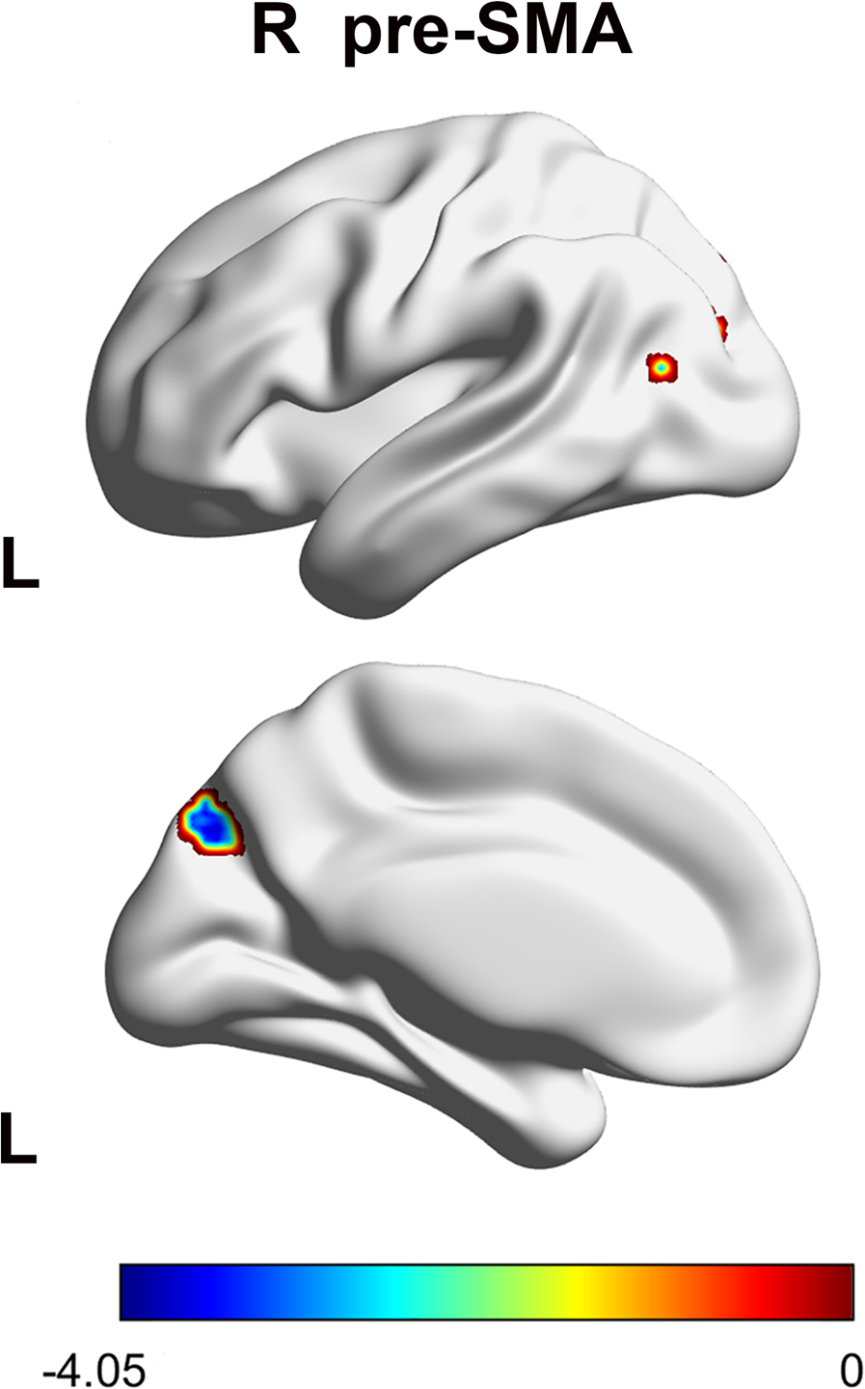
Sagittal views of the MNI brain areas showing reduced intrinsic functional connectivity (FC) with the preSMA in MWoAs compared to healthy controls. The surviving clusters were assigned thresholds at *p* < 0.001 (voxel level) and were FWE corrected to *p* < 0.05 at the cluster level

The patients with MWoAs showed a weaker FC between the R preSMA and the left precuneus (Table 2, Fig. 3).

**Fig. 4:**
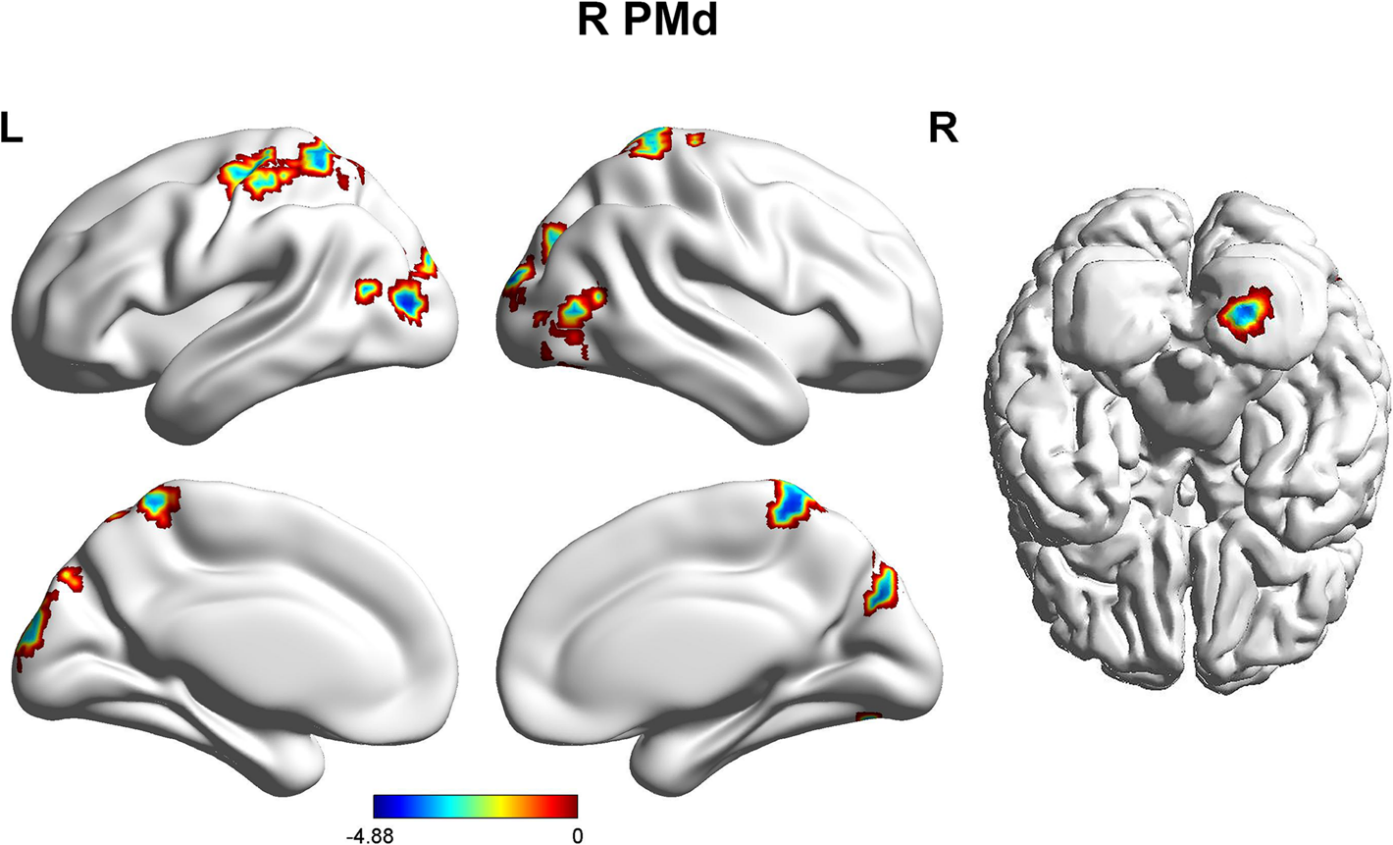
Sagittal views of the MNI brain areas showing reduced intrinsic functional connectivity (FC) with the PMd in MWoAs compared to healthy controls. The surviving clusters were assigned thresholds at *p* < 0.001 (voxel level) and were FWE corrected to *p* < 0.05 at the cluster level

The patients with MWoAs showed weaker FCs between the R PMd and the left precuneus, bilateral middle occipital gyrus, cerebellum areas, bilateral cuneus, and right postcentral gyrus (Table 2, Fig. 4).

### Correlations with clinical variables in migraine

**Fig. 5:**
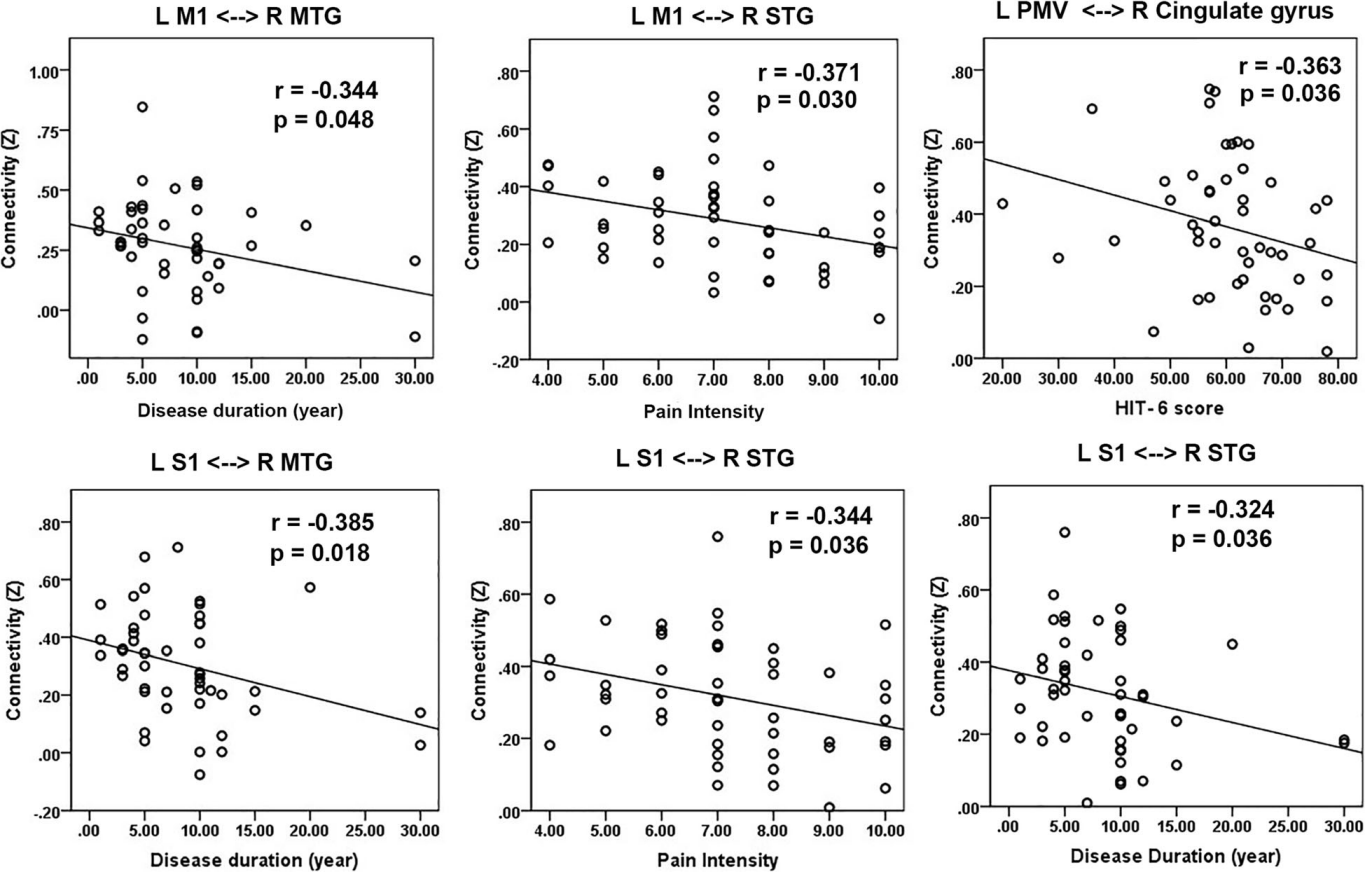
Scatter plot of clinical correlations of altered sensorimotor area functional connectivity (FC). Thresholds were *p* < 0.05, one-tailed, and FDR corrected for multiple comparisons

A negative correlation was found between the disease duration of the patients and the FC Z scores between the L M1 and the R middle temporal gyrus (MTG). The pain intensity of the patients was negatively correlated with the FC Z scores between the L SI and the R superior temporal gyrus (STG). The FC Z scores between the L S1 and the R MTG and R STG were also negatively correlated with disease duration in MWoAs. The HIT-6 score in MWoAs was negatively correlated with the FC Z score between the L PMv and the R cingulate gyrus (Fig. 5).

## Discussion

### Overview

Our previous study found that MWoAs exhibited functional abnormalities in the S1 and PMC and weaker FCs between the S1 and brain areas within the pain intensity and spatial regions involved in pain processing. In addition, we subdivided the sensorimotor areas as preSMA, SMA, PMd, PMv, M1, and S1 and found disrupted FCs between these sensorimotor brain regions and numerous other regions of the brain, such as the visual cortex, temporal cortex, posterior parietal lobule, prefrontal areas, precuneus, cingulate gyrus, sensorimotor areas proper and cerebellum areas. And we didn’t find any hyper sensorimotor connectivity in the MWoAs patients. These abnormal interactions between sensorimotor regions and multiple brain areas might contribute to dysfunction of the integration of information within and across the sensory modalities and, thus, could lead to the initiation of migraine attack and/or its associated symptoms. These impaired FCs were negatively correlated with clinical data, such as disease duration, pain intensity, and HIT-6 scores, which provided increased evidence that impaired FC between sub-regions of sensorimotor regions and multiple brain areas is involved in the pathophysiological mechanism of migraine.

### Functions of sensorimotor sub-regions

M1 is the main contributor to generating neural impulses that pass down to the spinal cord and control the execution of movement [21] and has been implicated in the ascending trigemino-thalamo-cortical nociceptive pathway [8]. Activation of S1 in studies of laser-evoked pain supports a role for S1 in the sensory aspects of pain, including discrimination and localization of pain intensity [22]. The SMA has many proposed functions, including the internally generated planning of movement, the planning of sequences of movement, and the coordination of the two sides of the body, such as in bi-manual coordination [23, 24]. The SMA is involved in pain anticipation [25]. The activity of pre-SMA neurons suggests that it involves a mechanism for switching from automatic to controlled eye movements [26]. The PMd may participate in learning to associate arbitrary sensory stimuli with specific movements or learning arbitrary response rules [27, 28]. The PMv is often studied with respect to its role in the sensory guidance of movement. Neurons here are responsive to tactile stimuli, visual stimuli, and auditory stimuli [29–31].

### The functional connectivity with S1

We found decreased functional connectivity between M1 and many other brain areas, such as the right middle occipital gyrus, right cuneus, right middle and superior temporal gyrus, left superior parietal gyrus, right postcentral gyrus, and right middle frontal gyrus, which are involved in pain perception and pain processing. A meta-analysis showed that excitatory M1 stimulation had a significant effect on reducing headache intensity and frequency of headache attacks in patients with migraine with a large effect size [11]. The middle occipital gyrus and right cuneus are involved in visual processing, and the migraineurs showed significantly higher activation than the control group in the left cuneus while viewing negative pictures [32]. The temporal pole participates in pain processing by mediating affective responses to painful stimuli and by acting as a multisensory integration zone responsible for processing painful, visual, auditory, and olfactory stimuli [3, 33, 34]. The posterior parietal lobule is now believed to underpin higher-order processes of sensory inputs, multisensory and sensorimotor integration, spatial attention, intention, and the conjoint representation of external space and the body [35]. The somatosensory cortex is contained in the postcentral gyrus, and a previous study found functional abnormalities in S1 and disrupted functional connectivity between S1 and other cortical regions [4]. The MFG, as part of the prefrontal cortex, is thought to be involved in the cognitive evaluation and modulation of pain [36]. The frequency of migraine attacks and the duration of the disorder had a significant impact on cortical thickness in the sensorimotor cortex and the middle frontal gyrus [37]. Disrupted FC between M1 and these brain regions might contribute to visual processing, multisensory integration, spatial attention and intention abnormalities, and dysfunction in the cognitive evaluation and modulation of pain.

### The functional connectivities with S1, SMA and preSMA

We found decreased functional connectivity between S1, the SMA and many other brain areas, such as the bilateral precuneus, bilateral postcentral gyrus, bilateral paracentral lobule, bilateral precentral gyrus, right cingulate gyrus, bilateral middle temporal gyrus, right superior temporal gyrus, bilateral inferior parietal lobule, right inferior and middle frontal gyrus, and left occipital superior gyrus and right cuneus. In addition, patients with MWoAs showed a weaker FC between the R preSMA and the left precuneus. The precuneus has been proposed to participate in information transfer and multimodal integration, which might be essential for the processing of spontaneous thoughts and for internal awareness [38]. The postcentral gyrus, paracentral lobule, and precentral gyrus are involved in sensorimotor networks, and a previous study found functional abnormalities and abnormal cortical thickness or gray matter volume in the sensorimotor network in migraineurs [4, 39, 40]. Gray matter volume decreases in the right anterior cingulate are related to the estimated frequency of headache attacks [41]. The temporal pole has been demonstrated to participate in pain processing and multisensory integration [3, 33, 34]. The inferior parietal lobule has been demonstrated to be involved in spatial discrimination and attention to pain [42–44]. The prefrontal region is thought to be involved in the cognitive evaluation and modulation of pain [36]. The middle occipital gyrus and right cuneus are involved in visual processing, and significantly increased cortical thickness was found in the lateral occipital cortex [45].

Brain areas with decreased functional connection to PMC.

### The functional connectivity with PMC

The bilateral PMCs were activated during a delayed match-to-sample task using thermal stimuli in healthy people; they receive input information from the ACC and contribute to the communication and selection of a decision about the nature of the afferent sensory information during both intensity and spatial discrimination [5]. The patients with MWoAs showed weaker FCs between R PMd and the left precuneus, bilateral middle occipital gyrus, cerebellum regions, bilateral cuneus, and right postcentral gyrus. The patients with MWoAs showed weaker FCs between the PMv and left precentral gyrus, right cingulate gyrus, bilateral precuneus, bilateral postcentral gyrus and L SMA. Numerous regions mentioned above are involved in the pathology of migraine. In addition, a previous study found structural changes in cerebellum-associated migraine [46]. The cerebellum has been demonstrated to be involved in human nociception [47] and is even suggested to play a modulating role in pain perception.

### Clinical correlations

The disease duration of the patients was negatively correlated with the FC Z scores between the L M1 and the R MTG and R STG and the FC Z scores between the L S1 and the R MTG and R STG, which suggests that recurrent headache attacks may drive functional changes and contribute to this disrupted network among the L M1, L S1 and temporal regions. The pain intensity of the patients was negatively correlated with the FC Z scores between the L SI and R STG. This result further indicates that the reduced FC between the L S1 and R STG is indeed a maladaptive functional plasticity closely related to pain intensity in MWoAs. The HIT-6 score in MWoAs was negatively correlated with the FC Z score between the L PMv and R cingulate gyrus, which suggests that the reduced FC is significantly related to the impact of recurrent migraine attacks on daily life.

### Limitation

Although our research revealed that MWoAs exhibited deficits in the functional connectivity between sensorimotor regions and other cortical areas, the current study had several limitations. First, we examined patients only in the interictal phase, and therefore, functional connectivity in the ictal phase must also be explored. Second, we focused on MWoAs but did not examine migraineurs with aura and chronic migraineurs. In a future study, we will evaluate the functional abnormalities in the sensorimotor regions of these other two populations during the ictal phase.

## Conclusion

We found that MWoAs exhibited disrupted functional connectivity between the sensorimotor areas and the visual cortex, temporal cortex, posterior parietal lobule, prefrontal areas, precuneus, cingulate gyrus, sensorimotor areas proper and cerebellum areas compared with healthy controls. These disrupted FCs might contribute to abnormalities in visual processing, multisensory integration, nociception processing, spatial attention and intention and dysfunction in cognitive evaluation and modulation of pain. Recurrent headache attacks might lead to the disrupted network between the L M1 and temporal regions and between the L S1 and temporal regions. Pain sensitivity and patient quality of life are closely tied to abnormal functional connectivity between sensorimotor regions and other brain areas.

## Data Availability

The datasets generated during and/or analyzed during the current study are available from the corresponding author on reasonable request.

## Abbreviations

ACC: Anterior cingulate cortex
fMRI: Functional magnetic resonance imaging
GM: Gray matter
HCs: Healthy controls
FC: Functional connectivity (FC)
FWE: Familywise error
FDR: False discovery rate
HMAT: Human Motor Area Template
HIT-6: Headache Impact Test
IPG: Inferior parietal gyri
MTG: Middle temporal lobule
MWoAs: Migraineurs without aura
MIDAS: Migraine Disability Assessment Scale
MNI: Montreal Neurological Institute
MRI: Magnetic resonance imaging
M1: Primary motor cortex
PAG: Periaqueductal gray matter
PMC: Premotor cortex
preSMA: Pre-supplementary motor area
PMv: Ventral premotor cortex
PMd: Dorsal premotor cortex
SMA: Supplementary motor cortex
S1: Primary somatosensory cortex
S2: Second somatosensory cortex
STG: Superior temporal gyrus
